# Acceleration-Based Method of Ice Quality Assessment in the Sport of Curling

**DOI:** 10.3390/s22031074

**Published:** 2022-01-29

**Authors:** Bartosz Dzikowski, Jerzy Weremczuk, Marek Pachwicewicz

**Affiliations:** 1 Warsaw University of Technology, Faculty of Electronics and Information Technology, Institute of Electronic Systems, Nowowiejska 15/19, 00-665 Warsaw, Poland; j.weremczuk@ise.pw.edu.pl; 2 MKTronic Sp. z o.o., Puławska 14, 02-512 Warsaw, Poland; marek@mktronic.pl

**Keywords:** curling, inertial measurements, sports measurements, curling ice, inertial measurement unit

## Abstract

Despite the significant influence of ice conditions on results in the sport of curling, players and ice technicians lack a measurement device that would objectively measure ice quality during a curling competition. This paper presents such a new measurement method by using a device consisting an inertial measurement unit (IMU) attached to the handle of the curling stone and data processing software. IMU is used to measure the vibration of curling stone during its movement on the surface of the ice. The acceleration signal is recorded, and then the software calculates the value of so-called *R* parameter in frequency domain. The value of *R* allows one to determine if an ice sheet had been pebbled and if the shape of pebbles is suitable for the game of curling. The presented system was tested in various ice conditions—on both freshly prepared and used ice. Ice technicians and players may use the proposed system to decide whether the ice surface is suitable for play or if it should be remade.

## 1. Introduction

Curling is a worldwide popular winter Olympic team sport, in which athletes slide 20-kilogram granite stones over the flat surface of ice. The field of play (sheet) is 45-meters long and 5-meters wide. On both ends of the sheet, colored rings called the house are the targets for players. A game consists of 8 or 10 ends and features two four-person teams (or one male and one female per team in mixed doubles format). After each end (which features 16 stones and 8 per team), a point is awarded for each stone that is closer to the center of the house (called tee) than any stone of the opposition [1].

During delivery, players set the stone in direct and rotational motion. Direct speed may be up to 4–5 m/s, and imposed angular speed is usually between 50 and 150 °/s. Rotation makes the stone follow a curved trajectory, and the lateral displacement in the stone’s path is called the curl. Curl direction depends on the direction of rotation—clockwise rotation makes the stone turn right and vice versa. Kameda has shown that the amount of curl mostly depends on the roughness of the curling stone’s running band [2]. This fact is widely used by icemakers, who often scratch running surfaces of stones before major events.

The physics behind curling stone movement is still not fully described, and new models are being proposed. Recently proposed models include front-back asymmetry models (assuming an asymmetry of friction forces at the front and the back of a running curling stone) [3,4,5,6,7,8,9,10,11] and pivot-slide models (taking that the curling stone pivots around specific points in the ice during its movement) [12,13]. Currently, no model is widely accepted, and scientists’ discussions about various models are still ongoing [14,15,16,17,18,19]. Regardless of proposed models, it is evident for players that preparation of the field plays an essential role in the game of curling. Unprepared or badly prepared ice may strongly influence competition or even make it impossible to play. Appropriate conditions in the field of play enable athletes to showcase their abilities and ensure fair competition.

Curling ice must be level (any unevenness would cause stones to drift to one side), but its surface must not be flat. Droplets of water must be sprayed on the ice to make it possible to play the game. After freezing, they form so-called pebbles that reduce the contact area between the ice and the bottom of the stone. However, these droplets form sharp cones on the ice, which is not desirable either because curling stones would crush these sharp cones. This is why pebble tops must be cut off with a knife (ice must be “scraped”), and only that makes it possible for the stones to slide in the field of play with ease.

The three ice structures are presented in Table 1. Sharp, uncut pebbles are observed during the ice preparation phase (A), and pebbled and scraped ice is observed during the competition (B). Flat ice may be observed after a very long match without maintenance or on a completely unprepared field of play (C).

Despite the important influence of ice conditions on game result, curlers and ice technicians lack a measurement device that would objectively measure the quality of ice to ensure a fair game. This research aims to investigate whether an accelerometer attached to the stone handle and data processing software can be used to differentiate between the three abovementioned structures (A, B, and C).

## 2. Measurements

### 2.1. Method

**Figure 1:**
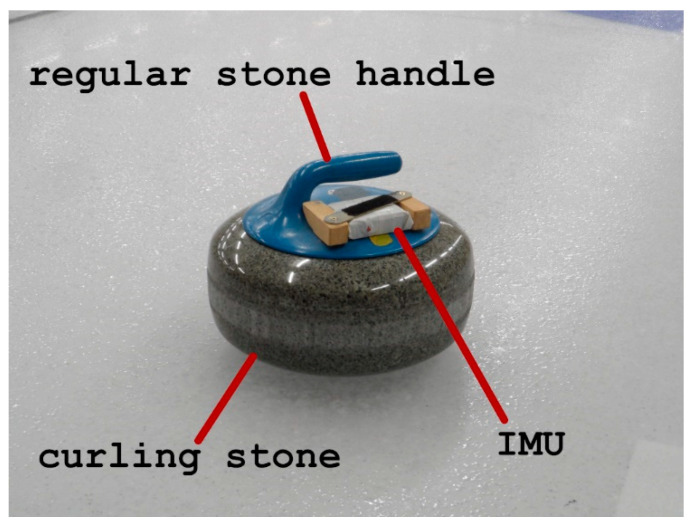
Curling stone with IMU.

A MEMS (microelectromechanical system) inertial measurement unit (IMU) with a 3-axis accelerometer was attached to the stone’s handle (Figure 1). The stone must have been cooled down before the experiment to avoid ice damage and produce reliable results. That is why a regular stone handle was replaced with a handle with IMU attached. A curler was asked to deliver the stone by playing a draw shot. The measurements were saved in IMU’s memory and copied to PC for analysis upon measurement completion. The device was attached to the horizontal, bottom part of the stone handle using wooden blocks (glued to the handle) and screws. A similar method was used in previous research by authors [20]. IMU weighs 80 g, which is omittable compared to the weight of a curling stone (20 kg).

In this experiment, the sampling frequency of IMU was set to 400 Hz. The sampling frequency was chosen based on IMU bandwidth (550 Hz). Collected acceleration samples were analyzed in the time and frequency domain (which is described in the next paragraph).

**Figure 2:**
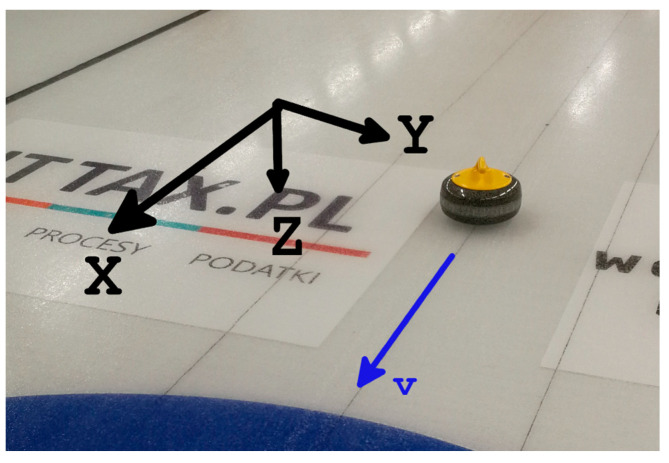
Axes as defined in the experiment.

In this experiment, the *X*-axis was defined as parallel to the curling sheet length, pointing from the start point to the target. The *Z*-axis was defined as pointing downward, and the *Y*-axis was defined accordingly to obtain a left-handed coordinate system (Figure 2).

**Figure 3:**
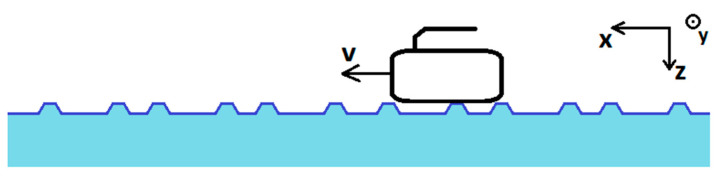
Curling stone traveling on pebbles (not in scale). The local coordinate system rotates with the stone. The global coordinate system is pictured above and is tied to the sheet of ice.

During its travel on the ice, the bottom of the curling stone touches the tops of pebbles and not the flat ice surface below pebbles (Figure 3). This fact makes the stone vibrate during the motion, and this vibration is measured in the experiment.

The character of vibration depends on the structure of the ice surface. During ice pebbling, water droplets are sprayed on the flat ice surface. They are not uniform; thus, some pebbles are formed taller and some are shorter. If a stone travels on such pebbled surfaces and not on nipped ice surfaces, it only comes in contact with the tallest pebbles. After nipping tops of pebbles, their height is much more uniform; thus, the stone running surface hits much more pebbles. Hence, we propose a hypothesis that the spectrum of stone vibration shifts towards higher frequencies on nipped ice compared to freshly pebbled ice.

### 2.2. Hardware

The device used in the experiment was developed by authors and consists of four main functional blocks:

- Power system;
- Microcontroller;
- Data storage;
- Sensors.

**Figure 4:**
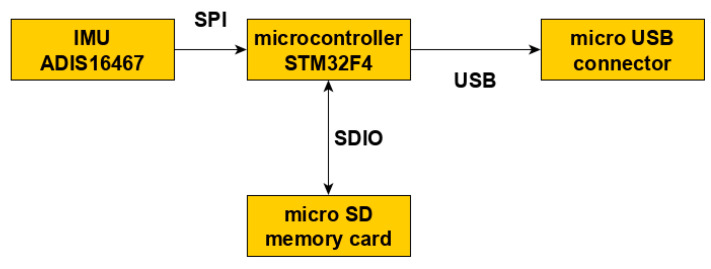
Block diagram of the device used in the experiment.

A block diagram of the device is presented in Figure 4. An Inertial Measurement Unit produced by Analog Devices (ADIS 16467) was used in this experiment. IMU features two sensors: a triaxial accelerometer (range ±40 g) and a triaxial gyroscope (range ±500 °/s) with temperature correction. These sensors have their own signal conditioning paths, autocalibration procedures, and dynamic compensation formulas, ensuring that data meet sensor specifications [21]. The signal of both gyroscope and thermometer were recorded as well but were not considered in detailed research. A gyroscope signal was used to choose the right part of the accelerometer signal for further analysis.

A microSD card is used for data storage. Saved data are accessible through a USB connection. The data are saved in binary format; thus, an additional program for data decryption was developed by the authors. Its task is to convert the binary file saved on the memory card into a human-readable CSV file.

The primary energy source of the device is a lithium polymer single-cell battery of 1300 mAh capacity. The device can be recharged using a standard micro USB port. This connector is also used for data transfer from the PC. The microcontroller from the STM32F4 family is based on ARM architecture and is manufactured by STMicroelectronics. It is responsible for the main loop implementation—sensors readings and data storage.

Acceleration signal has already been observed in previous research [20].

The most critical metrological parameters of the developed measurement device are presented in Table 2. The chosen IMU chip assures a high level of accuracy for inertial measurements of the stone for a single throw. It also has immunity against potential overload caused by a stone hitting the ice or other objects. With a resolution of 32-bit, the ADIS 16,467 accelerometer has typical sensitivity of 1.9 × 10^−8^ g/LSB (Least Significant Bit).

The firmware used in the microcontroller was developed by the authors in C programming language. The main task of the firmware is to record the data. After interfaces and modules initialization, the main loop of the program is started. Data from sensors are read every 2.5 ms (to achieve 400 Hz sampling frequency) and buffered in RAM. Every second, buffered data are written to a file on an SD card. The file can be read on a PC using a standard socket. Data processing was performed on a PC using MATLAB software.

## 3. Results

Measurements were taken at Curling Łódź, a state-of-the-art four-sheet curling rink located in Łódź, Poland. The stone used during the experiment came from one of the Olympic sets that are used in all top-level competitions around the world. During a training session, a competitive curler was asked to deliver the stone with IMU. No mechanical device was used for stone delivery.

**Figure 5:**
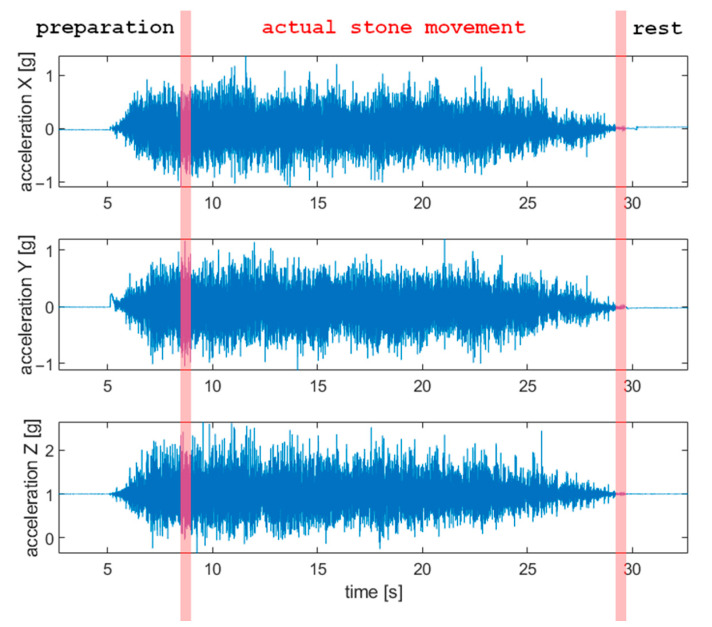
Results obtained with the accelerometer in the time domain (*f_s_* = 400 Hz). *X*-axis is horizontal and parallel to the longer dimension of the curling sheet, and *Y* is horizontal and perpendicular to *X*, and *Z* axis is vertical.

Results obtained with the accelerometer during the movement of a curling stone are presented in Figure 5 (in the time domain). Actual stone movement is contained between two vertical red lines. Before that period, the player delivered the stone, and afterward, the stone stood still on the ice.

**Figure 6:**
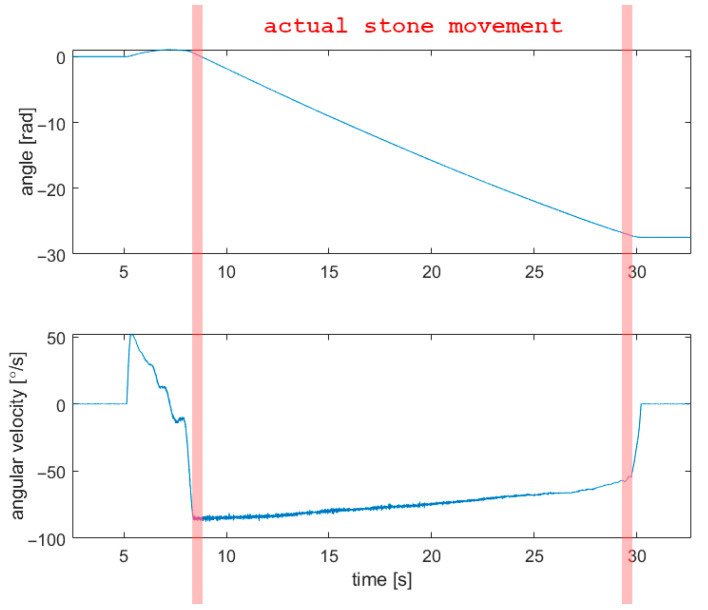
Results obtained with the gyroscope in the time domain (*f_s_* = 400 Hz).

Three periods mentioned above are clearly visible in gyroscope data, presented in Figure 6. Each shot had an initial angular velocity in the range of 50–100 °/s. During most of the curling shot, angular velocity decreases slightly, and most of the angular velocity is lost in the last 0.25 s. Lozowski observed similar behavior [22].

The measuring device was rotating with the stone itself; thus, to obtain acceleration parallel to the length of a curling sheet, gyroscope data was used according to Equations (1)–(3):

*a_X_* = *a_x_* cos *α* + *a_y_* sin *α*, (1)

*a_Y_* = −*a_x_* sin *α* + *a_y_* cos *α*, (2)

*a_Z_* = *a_z_*, (3)

where *x*, *y*, *z* are defined in the measuring device coordinate system, and *X*, *Y*, and *Z* are defined in the ice sheet coordinate system. *α* is the angle between *X* and *x*.

**Figure 7:**
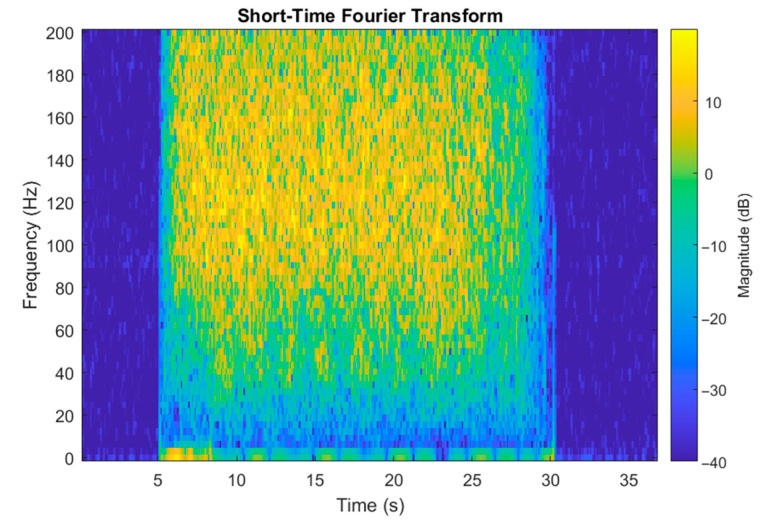
Spectrogram of obtained accelerometer signal. The used window length is 1 s, and window overlap is 75%.

**Figure 8:**
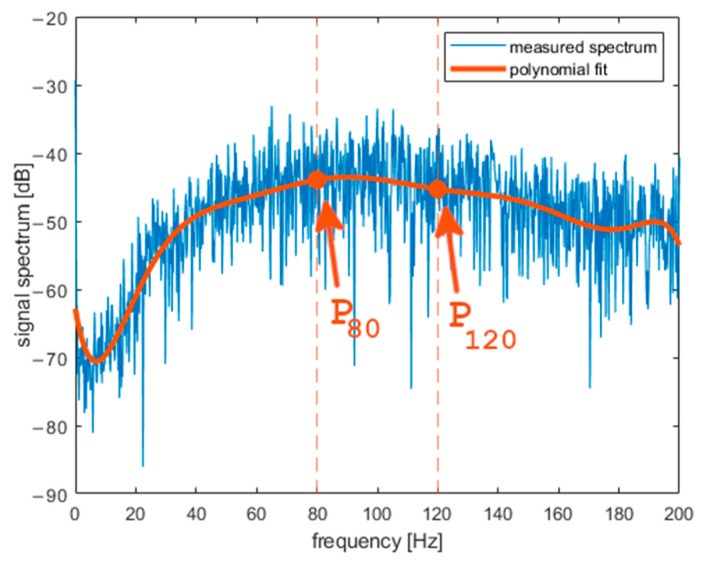
Results obtained with the accelerometer on freshly pebbled ice (frequency domain).

**Figure 9:**
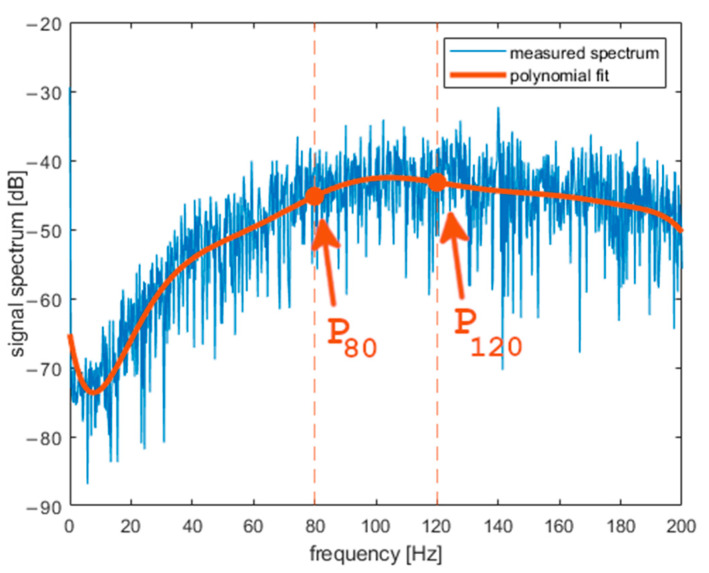
Results obtained with the accelerometer on pebbled and scraped ice (frequency domain).

**Figure 10:**
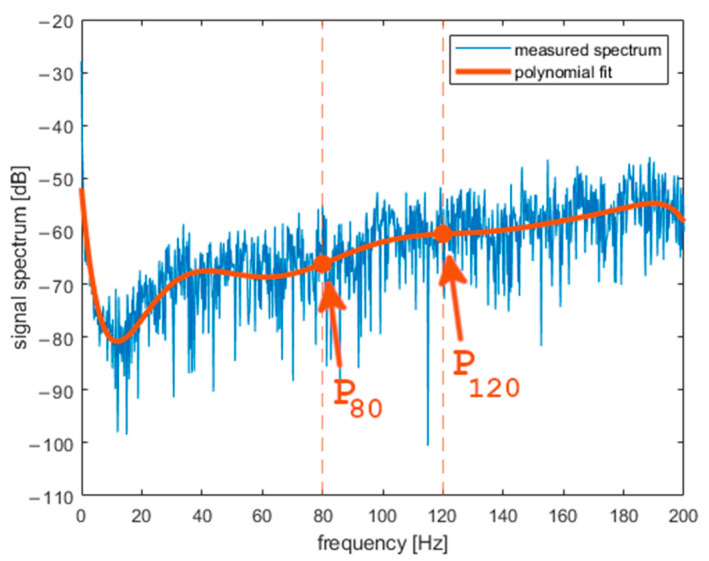
Results obtained with the accelerometer on flat ice (frequency domain).

**Figure 11:**
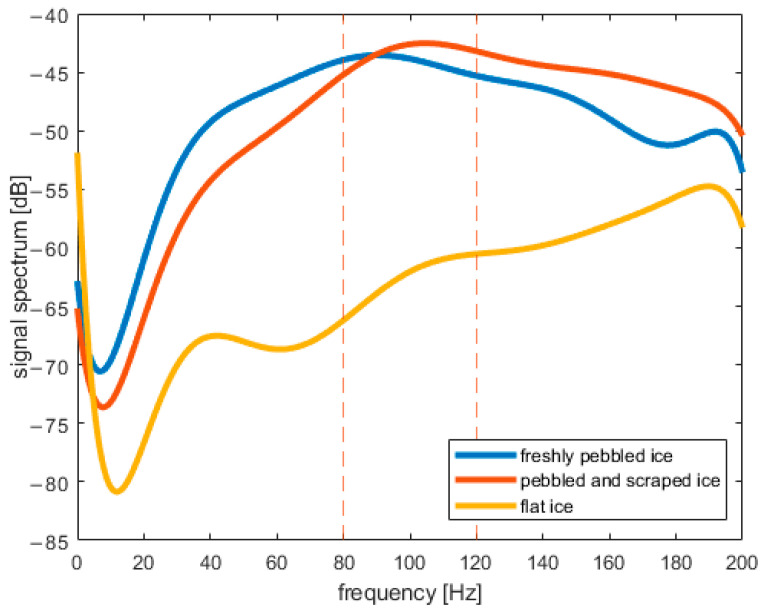
Three polynomial fits (from Figure 8, Figure 9 and Figure 10) presented on one chart.

*a_X_* was analyzed in the frequency domain after Fourier transform (FFT algorithm in MATLAB) was performed on it. Figure 7 presents the spectrogram of *a_X_*. The obtained spectrum does not change rapidly during the middle part of curling stone movement. One can observe fading of the signal during the last 5 s both in the frequency domain and in the time domain. In further analysis, we will use a signal portion from the middle of a shot. Figure 8, Figure 9 and Figure 10 present the spectrum of horizontal parallel acceleration obtained during a 5-second period (2000 samples) and its polynomial fit. The polynomial degree was 9, and it was fitted using the least square method to provide a smoothed version of the signal spectrum. It is clear that the signal obtained on pebbled ice has more power in the frequency region of 80–100 Hz than the signal recorded on flat ice. Generally, the character of the spectrum depends on ice quality. Three polynomial fits are presented together in Figure 11. The proposed physical explanation is that the stone hits only the highest pebbles on freshly pebbled ice. These pebbles are not as common. After nipping, more pebbles are struck by the rock as pebble height is more uniform, and the acceleration signal shifts towards higher frequencies. The signal is much weaker on flat ice as hitting pebbles does not happen.

**Figure 12:**
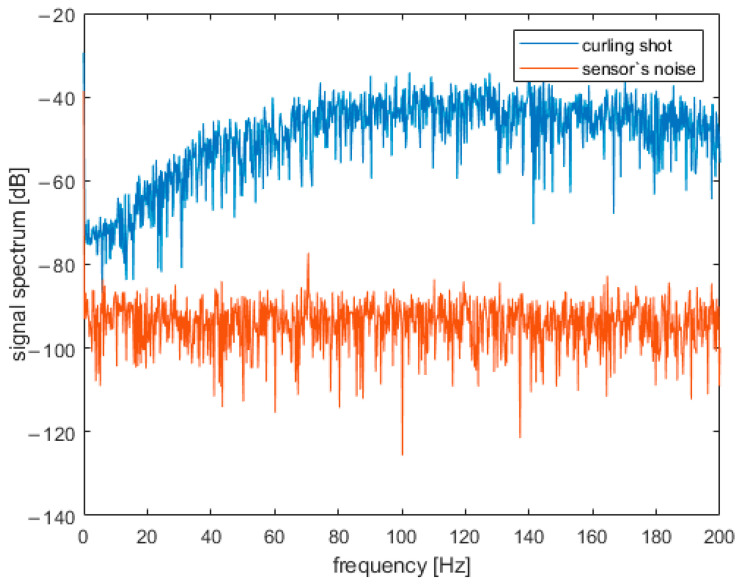
Signal acquired during a curling shot compared to acceleration sensor’s noise (frequency domain).

Signal acquired during a curling shot must be compared to the sensor’s noise. Figure 12 shows that the spectrum of the sensor’s noise (acquired when the sensor was not moving) is about 40–50 dB lower than the spectrum of the signal obtained during a curling shot. It shows that the nature of the observed signal is directly tied to the movement of a curling stone.

In order to compare spectra and types of ice objectively, parameter R was introduced. It is defined as follows.

*R* [dB] = P_120_ [dB] − P_80_ [dB] = P (120 Hz) [dB] − P (80 Hz) [dB], (4)

**Figure 13:**
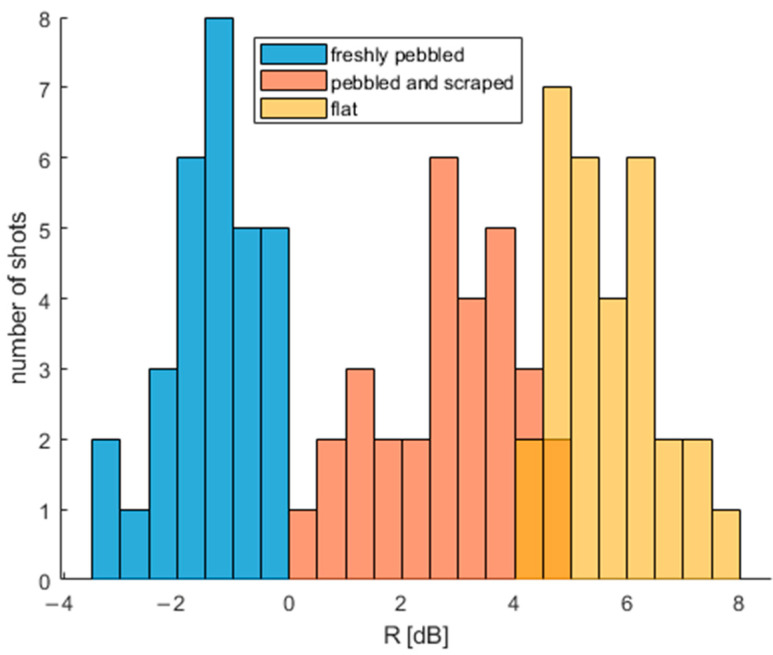
Histogram of *R* parameter for different types of ice and various shots.

*R* was calculated for 30 curling shots on each type of ice, and each obtained sample set was tested for normality using the Lilliefors test in MATLAB. In order to perform the test, data were converted back to a linear scale (instead of using results expressed in dB). The significance level was set to 0.05, and the null hypothesis stated that the dataset was normal. The Lilliefors test proved that obtained datasets might be treated as normal distributions (see Table 3). Thus, parametric measures will be used to describe the data. The obtained results are presented in Table 4 and Figure 13. Mean and standard deviation calculations were performed on a linear scale and converted to decibels afterward. The range of parameter values for different types of ice do not overlap within one standard deviation.

In order to compare three obtained distributions, a Welch *t*-test was performed in MATLAB with a null hypothesis that each pair of dataset came from distributions with different mean values. The significance level was set to 0.05. The test proved that the datasets came from three distributions with different mean values.

### Stability of Results within Curling Shot

**Figure 14:**
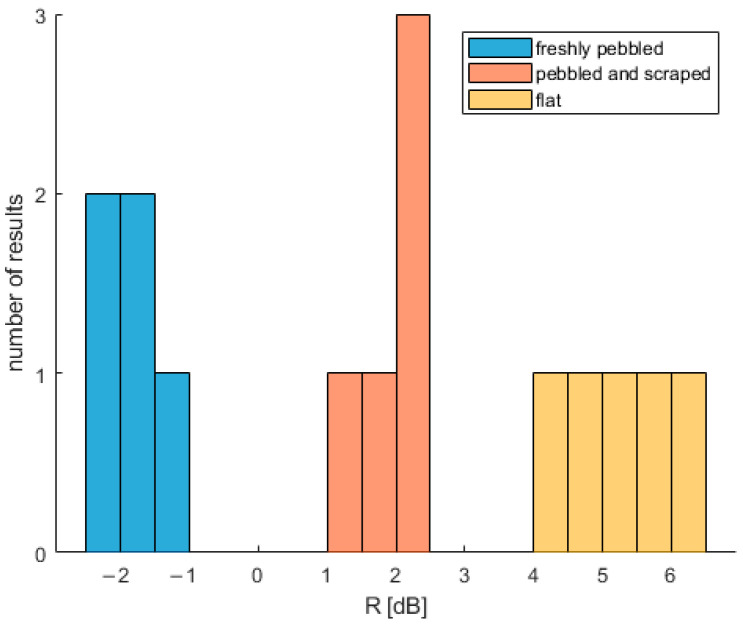
Histogram of *R* parameter for different parts of one curling shot.

The presented accelerometer results allow one to determine whether curling ice is suitable for play. However, how parameter R changes with time during a curling shot must also be analyzed. The values of *R* calculated for different parts of one curling shot per ice type of ice are presented in Table 5. The parts are 5 s periods (2000 samples) starting at different time points during the stone’s journey. The value of *R* does vary within each shot, but it never leaves the region associated with the particular type of ice (Figure 14).

## 4. Discussion

Several scientific papers in the field of curling try to explain the mechanism that induces a curl (lateral motion) in the stone [2,3,5,6,7,8,9,10,11,12,13,19,23,24]; moreover, the papers attempt to analyze the effect of sweeping on the stone motion [25,26,27]. However, to the authors’ best knowledge, no attempt has been made to develop a method to assess curling ice quality objectively.

Inertial measurement units are used in sports measurements [28,29,30]. IMU mounted to the handle of a curling stone was previously used by Lozowski [22]. However, in that research study, the IMU was used to measure the macroscopic movement of a stone, and vibrations (resulting from the interaction of stone and ice) were filtered out and were not analyzed. Lozowski compared the obtained results with a numerical model of curling ice friction and dynamics published in [23] and demonstrated that the “ordinary friction” model is unable to predict any curl (lateral motion of the stone). Lozowski observed similar behavior of stone’s angular velocity as presented in Figure 6 (slow deceleration for most of the curling stone’s movement and rapid deceleration at the end). This manuscript presents a different, novel approach of using an IMU to analyze the vibrations of a curling stone.

In this manuscript, a new, objective acceleration-based method was proposed to assess curling field of play (ice) quality during the curling competition. The procedure is based on signal analysis from the accelerometer attached to the handle of a moving curling stone. In the studies, it was found that the sensor signal acquired during a curling shot is about 40–50 dB higher than the sensor’s noise. It confirms that IMU range and resolution was correctly selected, and results of short-time analysis of signal frequency spectra could be used to characterize curling stone vibration during movement. Parameter *R* was proposed to characterize the ice state (freshly pebbled, pebbled and scraped, and flat). Changes in *R*-value can be used to monitor the quality of ice during curling competitions continuously. It was also confirmed that the value of *R* is stable for the all stone movements during a draw shot and does not vary significantly between repeated attempts on the same ice conditions.

The experiments were conducted only on one rink. Further research is necessary to determine whether the absolute value of *R* varies from one ice rink to another. If confirmed, a correction formula on *R*-value should be elaborated (e.g., a new reference frequency set for calculation of *R* value should be developed).

The method proposed in this manuscript may be easily applied by ice technicians and players to evaluate the state of curing ice during competition and practice. It may indicate whether the ice surface is suitable for the game and when it should be remade to assure fair play. The method is fast, as curling stone delivery usually takes about 20–30 s and is easy to apply. It could be used in between end breaks during a curling game (which lasts for 1 to 5 min). The electronic recording device is small and light and does not change the trajectory of a granite curling stone.

It is also worth considering extending the device functionality with wireless communication with PC (e.g., Bluetooth), improving sample transfer.

However, the proposed method also has some limitations. Most likely, it would not be usable in an ice rink with high air humidity (e.g., without a dehumidifying system). In such a case, water is condensed on the ceiling and then falls onto the ice as huge droplets, changing the shape of the ice surface. It is also unknown how frost deposition on the ice would influence results.

## Figures and Tables

**Table 1:** Types of observed curling ice (a coin is put in the ice to fix the photo scale; coin diameter is 15.5 mm).

Type of Ice	Photo	Schematic Structure	Comment
A—pebbled ice (sharp pebbles)	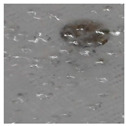	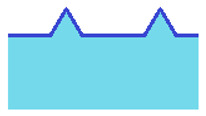	observed during preparation (not suitable for play)
B—pebbled and scraped ice	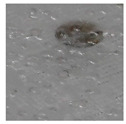	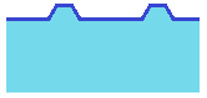	observed during the competition (suitable for play)
C—flat ice surface	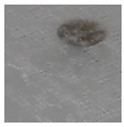	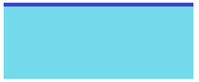	observed after a very long competition without maintenance or unprepared (not suitable for play)

**Table 2:** Selected metrological parameters of IMU ADIS16467 [21].

Parameter	Value
ACCELEROMETER	
Range	40 g
Sensitivity	52,428,800 LSB/g
In run bias stability	13 µg
Velocity random walk	0.037 m/s/√h
GYROSCOPE	
Range	500 °/s
Sensitivity	40 LSB/°/s
In run bias stability	2.5 °/h
Angular random walk	0.15 °/√h

**Table 3:** The results of Lilliefors normality test for obtained data sets.

Type of Ice	Freshly Pebbled Ice	Pebbled and Scraped Ice	Flat Ice
*p*-value	0.36	0.48	0.25
Critical value	0.16	0.16	0.16
Test decision for the null hypothesis	not rejected	not rejected	not rejected

**Table 4:** Values of parameter *R* in various ice conditions.

Type of Ice	Freshly Pebbled Ice	Pebbled and Scraped Ice	Flat Ice
*R* mean value—standard deviation	−2.14 dB	1.59 dB	4.68 dB
*R* mean value	−1.32 dB	2.84 dB	5.72 dB
*R* mean value + standard deviation	−0.57 dB	3.94 dB	6.65 dB
Number of shots	30	30	30

**Table 5:** Values of *R* parameter for different parts of one curling shot.

	*R* on Freshly Pebbled Ice	*R* on Pebbled and Scraped Ice	*R* on Flat Ice
shot 1, part 1	−1.36 dB	2.01 dB	5.66 dB
shot 1, part 2	−2.07 dB	1.81 dB	5.12 dB
shot 1, part 3	−1.56 dB	2.22 dB	6.27 dB
shot 1, part 4	−1.81 dB	1.03 dB	4.62 dB
shot 1, part 5	−2.18 dB	2.34 dB	4.47 dB

## Data Availability

Not applicable.
